# Long-term prognostic factors of chronic central serous chorioretinopathy after half-dose photodynamic therapy: A 3-year follow-up study

**DOI:** 10.1371/journal.pone.0181479

**Published:** 2017-07-24

**Authors:** Fuminori Haga, Ruka Maruko, Chiaki Sato, Keiko Kataoka, Yasuki Ito, Hiroko Terasaki

**Affiliations:** Department of Ophthalmology, Nagoya University Graduate School of Medicine, 65 Tsuruma-cho, Showa-ku, Nagoya, Japan; Massachusetts Eye & Ear Infirmary, Harvard Medical School, UNITED STATES

## Abstract

**Purpose:**

To evaluate the long-term efficacy and factors involved in the recurrence and persistence of subretinal fluid (SRF) after half-dose photodynamic therapy (PDT) for chronic central serous chorioretinopathy (CSC).

**Methods:**

In this retrospective observational case series, 79 eyes (73 patients) with chronic CSC were treated with half-dose PDT and followed up for at least 3 years. They were divided into successful (64 eyes) and unsuccessful (15 eyes) groups based on SRF absorption and disease recurrence after one PDT session. Age, best-corrected visual acuity (BCVA), central foveal thickness, neuroretinal thickness, height of SRF, subfoveal choroidal thickness, window defect area detected by fluorescein angiography, and PDT spot area were compared between the groups. Factors associated with PDT success and BCVA at 3 years were investigated.

**Results:**

LogMAR BCVA improved from 0.21±0.24 to 0.08±0.16 (*P*<0.001) at 3 years after PDT. Compared with the unsuccessful group, the successful group had a significantly younger mean age (50.5±9.7 vs. 56.5±9.1 years, *P* = 0.032) and better baseline BCVA (0.18±0.23 vs. 0.32±0.25, *P* = 0.034). Other parameters were not significantly different. Multivariate analyses showed that unsuccessful PDT was significantly associated with lower baseline BCVA (*P* = 0.026) and older age (*P* = 0.029) and that BCVA at 3 years after PDT was positively associated with baseline BCVA (*P*<0.001).

**Conclusions:**

Half-dose PDT has a long-term efficacy in chronic CSC. Relatively early PDT may improve anatomic and functional outcomes of chronic CSC.

## Introduction

Central serous chorioretinopathy (CSC) is characterized by the accumulation of subretinal fluid (SRF) in the macula, and it frequently affects young-to-middle-aged men.[1] The risk factors of CSC include the use of corticosteroid medications,[2, 3] pregnancy,[4] psychological stress,[1, 5] type A personality,[6] smoking,[7] and male gender.[8] Indocyanine green angiography (ICGA) has shown the hyperpermeability of choroidal vessels in eyes with CSC,[9] and optical coherence tomography (OCT) has shown that the choroid is thicker in eyes with CSC than in normal eyes.[10] The exact etiology of CSC is unknown, although disturbances in choroidal circulation are suggested to be related to CSC. CSC is generally recognized as a self-limiting disease with good prognosis, and it usually resolves spontaneously within 3–4 months. However, in the long term, approximately half of the patients experience persistent or recurrent SRF.[11, 12] In these patients, the prognosis can be poor due to complications such as diffuse atrophy of the retinal pigment epithelium (RPE), subretinal fibrosis, and thinning of the outer sensory retina.[13]

Laser photocoagulation (LP) is generally used to treat CSC that does not spontaneously resolve. However, CSC with a subfoveal or parafoveal leakage point is difficult to treat with LP because of the complication of scotoma. Chronic CSC with broad and indistinct leakage cannot be treated with LP because of the indistinct leakage point. In 2003, photodynamic therapy (PDT) with full-dose verteporfin was reported as a new treatment for chronic CSC.[14] The reduction of SRF in CSC after PDT is theorized to be caused by short-term choriocapillary hypoperfusion and long-term choroidal vascular remodeling.[14] PDT can be administered for CSC with a subfoveal or parafoveal leakage point and for chronic CSC with broad and indistinct leakage. It has been shown to be effective in reducing SRF and improving visual acuity in chronic CSC.[15–18] However, complications such as secondary choroidal neovascularization (CNV), pigmentary changes of the RPE, and persistent choroidal ischemia can occur after PDT with full-dose verteporfin.[14, 16, 19, 20] In recent years, PDT with half-dose, instead of full-dose, verteporfin has been conducted in chronic CSC with similar effects and reduced complications.[21–26]

Although the long-term outcomes of half-dose PDT for chronic CSC have been reported,[24, 25]the long-term prognostic factors are unknown. The purpose of this study was to determine the long-term prognostic factors of chronic CSC after half-dose PDT. We investigated the factors related to the recurrence and persistence of SRF in chronic CSC after half-dose PDT. We also assessed the factors related to the visual acuity of chronic CSC at 3 years after half-dose PDT.

## Methods

### Study participants

We retrospectively reviewed the medical records of chronic CSC patients who have received half-dose PDT from July 2009 to May 2012 at the Department of Ophthalmology, Nagoya University Graduate School of Medicine. CSC was defined as detachment of the neurosensory retina from the macula caused by idiopathic leakage or diffuse leakage from the RPE. Leakage from RPE was detected by fluorescein angiography (FA) and choroidal vascular hyperpermeability was detected by ICGA. Chronic CSC was diagnosed when symptoms of SRF persisted for more than 3 months. The inclusion and exclusion criteria are listed in Table 1. This study was approved by the Institutional Review Board of Nagoya University Graduate School of Medicine. Written informed consent was obtained from all patients. The procedures of this study followed the tenets of the Declaration of Helsinki.

**Table 1:** Inclusion and exclusion criteria of the patients recruited in the study.

**Inclusion criteria**
・ Patients whose follow-up period was available for more than three years after half-dose PDT ・ Persistence of subfoveal fluid on OCT or other subjective symptoms for three months or more ・ Presence on FA of active angiographic leakage secondary to CSC ・ Choroidal vascular hyperpermeability and abnormal dilation of choroidal vasculature, consistent with a diagnosis of CSC ・ Difficult to treat by LP because of leakage from the subfovea, parafovea, or broad area
**Exclusion criteria**
・ Presence of CNV or other maculopathy ・ Presence of choroidopathy that may affect choroidal thickness ・ Other ocular diseases that could affect visual acuity ・ Previous treatment by STTA injections and intravitreal anti VEGF injections ・ Previous LP within 3 months before half-dose PDT for CSC ・ Previous PDT for CSC ・ Optic media opacity that could interfere with adequate acquisition of OCT, FA, and ICGA images ・ Absolute contraindication for half-dose PDT or FA ・ Myopia(≤6D), hyperopia(>6D) and aphakia

PDT, photodynamic therapy; OCT, optical coherence tomography; CSC, central serous chorioretinopathy; CNV, choroidal neovascularization; LP, laser photocoagulation; STTA, sub-tenon triamcinolone acetonide: VEGF, vascular endothelial growth factor; FA, fluorescein angiography; ICGA, indocyanine green angiography

### Photodynamic therapy

All patients received half of the standard dose of verteporfin (3 mg/m^2^), which was infused over 10 min, followed by laser treatment 15 min after. The laser was delivered to cover the area of choroidal hyperpermeability that was detected during the middle phase of ICGA. The total light energy was 50 J/cm^2^. After treatment, patients were instructed to avoid sunlight for 5 days.

### Baseline and follow-up examination

Patients were assessed at baseline and at 1, 3, 6, 12, 24, and 36 months after PDT. Patients were evaluated based on age, gender, previous or concurrent use of corticosteroids, and history of smoking at initial visit. Measurements of best-corrected visual acuity (BCVA), slit-lamp examination, dilated fundus examination, and OCT were performed at each visit. BCVA was measured based on the Japanese standard decimal visual acuity chart at 5 m and was converted to the logarithm of the minimum angle of resolution (logMAR) scale for analysis. OCT was performed with enhanced depth imaging (EDI-OCT) using spectral-domain OCT (Spectralis HRA+OCT, Heidelberg Engineering, Heidelberg, Germany). During post-PDT visits, the locations assessed at baseline were re-examined using the eye-tracking function of Spectralis OCT. OCT images were used to measure central foveal thickness (CFT), neuroretinal thickness (NRT), height of SRF, and subfoveal choroidal thickness (SCT). They were manually measured beneath the fovea on horizontal and vertical OCT images using the built-in caliper function of the Heidelberg Eye Explore software. The average of the horizontal and vertical scan measurements was used in evaluations.

CFT was defined as the distance between the internal limiting membrane and the outer portion of the RPE. Height of SRF was defined as the distance between the outer surface of the sensory retina and the inner portion of the RPE. NRT was calculated by subtracting SRF height from CFT. SCT was defined as the distance between the outer portion of the RPE and the inner surface of the sclera. Baseline FA and ICGA were performed using Spectralis HRA (Heidelberg Engineering, Heidelberg, Germany) in all patients. Window defect was defined as an area of hyperfluorescence on FA without an increase in size from early to late phase. All areas of the window defect on a 30-degree field of view were measured and summed up using the built-in caliper function of the Heidelberg Eye Explore software. Retreatment was considered if SRF persisted for over 1 year after PDT or if SRF recurred and persisted for 3 months.

### Study group characteristics

The eyes were assigned to two study groups based on the presence or absence of SRF following PDT. During the follow-up period after PDT, eyes with complete SRF absorption without recurrence were classified in the successful group, whereas eyes with incomplete SRF absorption or recurrence after complete SRF absorption were classified in the unsuccessful group.

### Statistical analysis

Statistical comparisons were made between the successful group and the unsuccessful group using Student’s *t*-test for numeric data and Fisher’s exact test for non-numeric data. Multiple logistic regression analysis was performed to investigate the factors that were significantly associated with unsuccessful PDT. Age, BCVA before PDT, NRT at baseline, SCT at baseline, PDT spot area, and window defect area were assigned as the independent variables for the analysis. Repeated analysis of variance (ANOVA) and Bonferroni post hoc test were used to compare the clinical data at baseline and at 1,3,6,12,24, and 36 months after PDT. SCT subgroup analysis was conducted between the PDT effective group (eyes with complete resolution of SRF within 12 months after PDT) and the PDT ineffective group (eyes with persistent SRF for 12 months after PDT). The PDT effective group included recurrence cases. Multiple regression analysis was performed to investigate the factors that were significantly associated with BCVA at 3 years after PDT. BCVA at 3 years after PDT was assigned as the dependent variable, whereas age, BCVA before PDT, NRT at baseline, SCT at baseline, PDT spot area, and window defect area were assigned as the independent variables for the analysis. Statistical analyses were performed using SPSS version 22.0 (SPSS Inc., Chicago, Illinois, USA). A *P* value <0.05 was considered statistically significant.

## Results

79 eyes of 73 chronic CSC patients were included in this study. Among the patients, 61 (84%) were men and 12 (16%) were women with a mean age of 51.7 ± 9.8 years (range, 33–74 years) at the time of PDT. The overall mean follow-up period was 50.1 ± 10.3 months (range, 36–74 months). There were 11 (14%) eyes with previous or concurrent exposure to corticosteroids, 53 (67%) eyes of patients with a history of smoking, and 4 (5.1%) eyes of patients who received LP before PDT. The mean PDT spot area was 12.4 ± 9.6 mm^2^ (range, 3.0–43.0 mm^2^).

**Fig 1:**
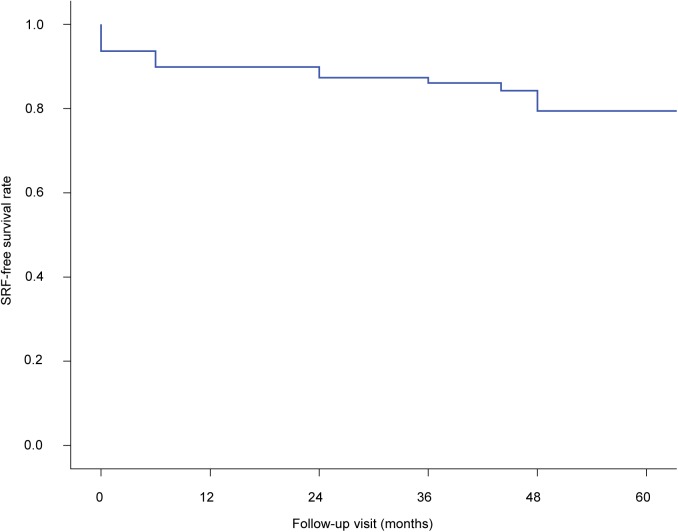
Kaplan–Meier survival plot showing the proportion of eyes without subretinal fluid after initial half-dose photodynamic therapy. Recurrence continued to occur during the 5-year follow-up period.

After initial PDT, absorption of SRF was complete in 74 (94%) and incomplete in 5 (6%); 10 (13%) eyes had SRF recurrence. Therefore, 64 (81%) were assigned to the successful group, and 15 (19%) were assigned to unsuccessful group. Recurrence occurred at an average of 30.6 ± 20.6 months (range, 6–64 months) after initial PDT. The SRF-free survival graph is shown in Fig 1. Of the10 eyes that had SRF recurrence, 8 underwent additional PDT and had complete SRF absorption, 1 had SRF that spontaneously resolved a few months later, and 1 eye was not treated because the patient refused further treatment.

The clinical characteristics of the two groups are summarized in Table 2. No significant differences were found between the two groups in terms of gender, previous or concurrent use of corticosteroids, history of smoking, refractive error, CFT at baseline, NRT at baseline, height of SRF at baseline, SCT at baseline, PDT spot area, and window defect area. However, age (*P* = 0.032) and BCVA before PDT (*P* = 0.027) showed statistically significant difference between the two groups.

**Table 2:** Comparison of baseline clinical characteristics according to success of half-dose PDT.

	Successful group (n = 64)	Unsuccessful group (n = 15)	*P* Value
Age (years)	50.5 ± 9.7	56.5 ± 9.1	.032^†^
Male, n (%)	53 (82.8)	13 (86.7)	>0.999^*^
Previous or concurrent use of corticosteroid, n (%)	7 (10.9)	4(26.7)	.206^*^
History of smoking, n (%)	46 (71.9)	7(46.7)	.074^*^
Refractive error (SE, diopters)	−0.4 ± 1.7	-0.5 ± 2.0	.940^†^
BCVA (logMAR)	0.18 ± 0.23	0.32 ± 0.25	.034^†^
Central foveal thickness (μm)	326.6 ± 87.9	298.9 ± 111.6	.302^†^
Neuroretinal thickness (μm)	179.5 ± 37.5	166.5 ± 22.2	.221^†^
Subretinal fluid height (μm)	146.3 ± 84.9	128.0 ± 114.6	.486^†^
Subfoveal choroidal thickness (μm)	431.3 ± 130.7	366.4 ± 100.1	.076^†^
PDT spot area (mm^2^)	12.6 ± 9.9	11.6 ± 8.5	.704^†^
Window defect area (mm^2^)	6.4 ± 8.9	6.3 ± 7.7	.969^†^

PDT, photodynamic therapy; SE, spherical equivalent; BCVA, best-corrected visual acuity; logMAR, logarithm of the minimal angle of resolution

Data are presented as mean ± standard deviation

^*^Fisher exact test

^†^Student *t-*test

### Best-corrected visual acuities

**Fig 2:**
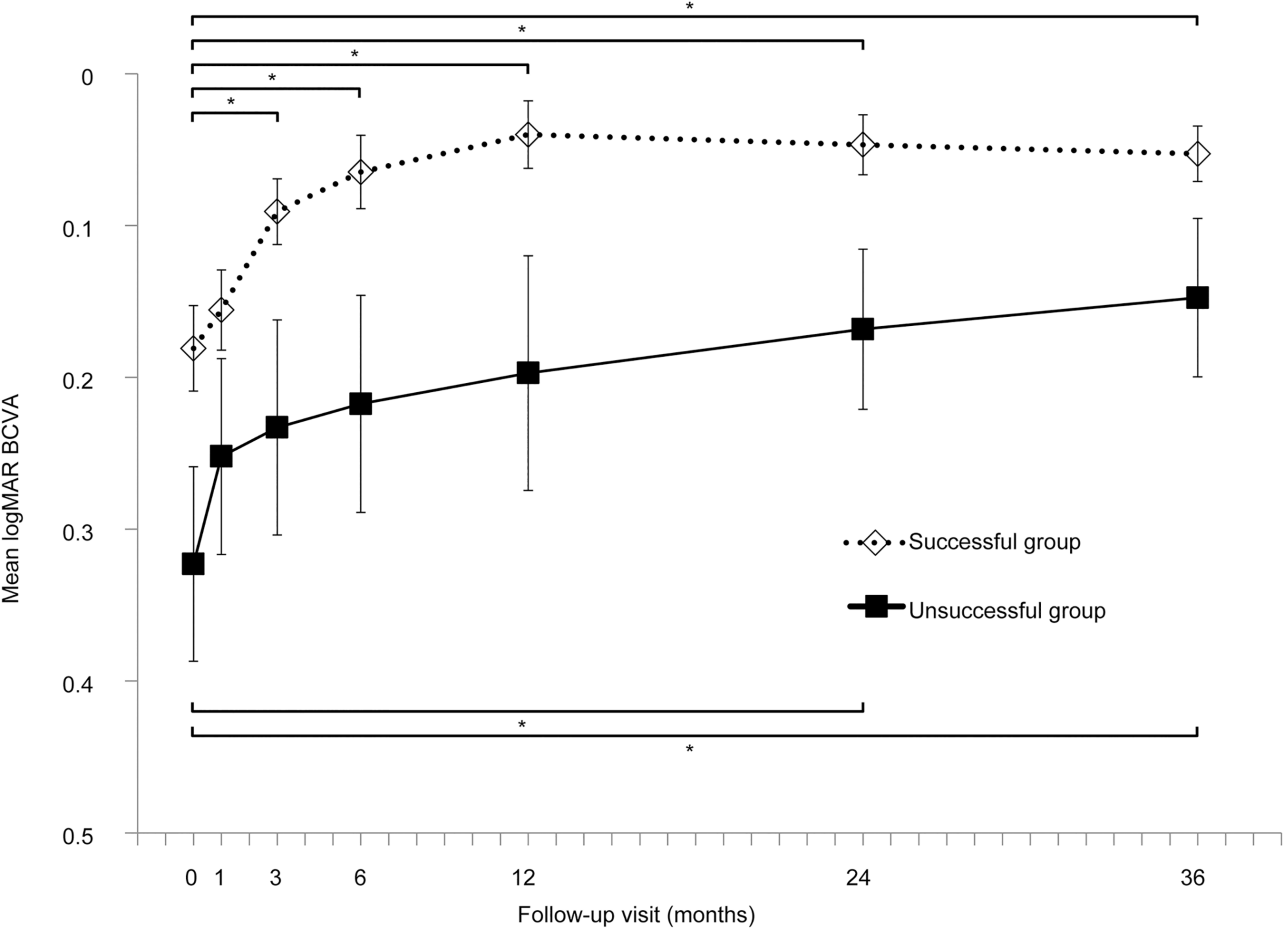
Long-term visual acuity changes following half-dose photodynamic therapy in eyes with chronic central serous chorioretinopathy. Baseline best-corrected visual acuity (BCVA) significantly improved after 3, 6, 12, 24, and 36 months in the successful group and at 24 and 36 months in the unsuccessful group. All values are presented as mean ± standard error of the mean. *P < 0.05.

The mean BCVA at baseline and at 1, 3, 6, 12, 24, and 36 months after PDT was 0.21 ± 0.24, 0.17 ± 0.22, 0.12 ± 0.20, 0.09 ± 0.22, 0.07 ± 0.21, 0.07 ± 0.17, and 0.08 ± 0.16, respectively. Baseline BCVA significantly improved at 6, 12, 24 and 36 months after PDT (all *P*<0.001). In the successful group, the BCVA at baseline and at 1, 3, 6, 12, 24, and 36 months after PDT was 0.18 ± 0.23, 0.16 ± 0.21, 0.09 ± 0.17, 0.06 ± 0.19, 0.04 ± 0.18, 0.05 ± 0.16, and 0.05 ± 0.15, respectively. Baseline BCVA significantly improved at 3, 6, 12, 24, and 36 months after PDT (all *P*<0.001) (Fig 2). In the unsuccessful group, the BCVA at baseline and at 1, 3, 6, 12, 24, and 36 months after PDT was 0.32 ± 0.25, 0.25 ± 0.25, 0.23 ± 0.27, 0.22 ± 0.28, 0.20 ± 0.30, 0.17 ± 0.20, and 0.15 ± 0.20, respectively. Baseline BCVA significantly improved at 24 and 36 months after PDT (*P* = 0.01, *P* = 0.003, respectively) (Fig 2). The BCVA values at each time point were compared between the successful and unsuccessful groups. BCVA in the unsuccessful group was significantly worse than that in the successful group at baseline (*P* = 0.034) and at 3 months (*P* = 0.013), 6 months (*P* = 0.014), 12 months (*P* = 0.009), 24 months (*P* = 0.013), and 36 months (*P* = 0.040) after PDT, but not at 1 month after PDT (*P* = 0.129).

### SCT changes after half-dose PDT

**Fig 3:**
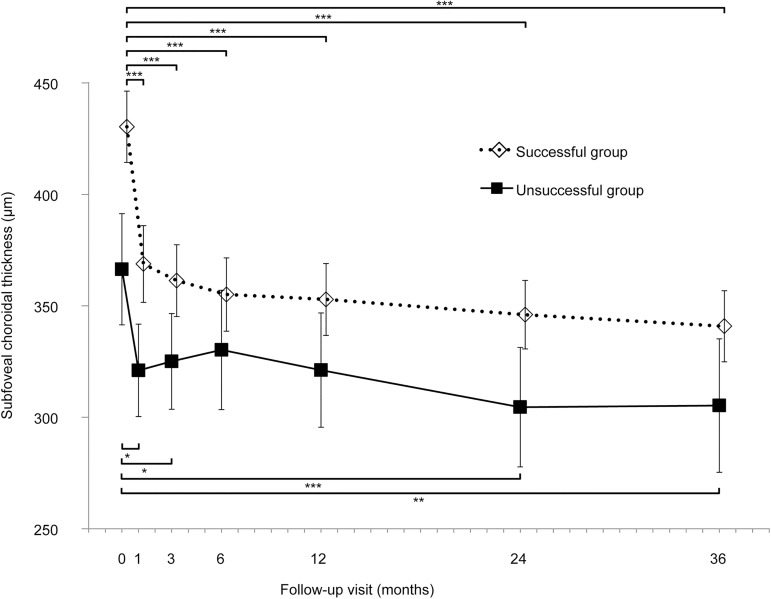
Long-term subfoveal choroidal thickness changes following half-dose photodynamic therapy (PDT) in eyes with chronic central serous chorioretinopathy. After PDT, baseline subfoveal choroidal thickness significantly decreased at each visit in the successful group and at 1, 3, 24, and 36 months in the unsuccessful group. All values are presented as mean ± standard error of the mean. *P < 0.05, **P < 0.01, ***P < 0.001.

In the successful group, the mean SCT at baseline and at 1, 3, 6, 12, 24, and 36 months after PDT was 428.7 ± 15.8μm, 365.6 ± 16.4μm, 359.5 ± 15.6, 352.2 ± 16.2, 350.9 ± 16.1, 346.0 ± 15.7, and 342.8 ± 16.3 μm, respectively. After PDT, baseline SCT became significantly thinner at 1, 3, 6, 12, 24, and 36 months (all *P*<0.001) (Fig 3).

In the unsuccessful group, the SCT at baseline and at 1, 3, 6, 12, 24, and 36 months after PDT was 366.4 ± 31.8μm, 321.0 ± 33.1μm, 325.1 ± 31.5μm, 330.2 ± 32.6μm, 321.2 ± 32.5μm, 304.5 ± 31.6μm, and 305.3 ± 32.9 μm, respectively. After PDT, baseline SCT became significantly thinner at 1 month (*P* = 0.005), 3 months (*P* = 0.023), 24 months (*P* <0.001), and 36 months (*P* = 0.001); however, baseline SCT was not significantly different at 6 months (*P* = 0.381) and12 months (*P* = 0.067) after PDT.

Subgroup analysis showed that in the PDT effective group, baseline SCT became significantly thinner at 3 months after PDT (423.2 ± 14.8 μm vs. 355.8 ± 14.6 μm, *P*<0.001). In the PDT ineffective group, baseline SCT did not significantly change at 3 months after PDT (344.4 ± 43.6 μm at baseline vs. 332.8 ± 38.2 μm at 3 months after PDT, *P* = 0.54). In the PDT effective group, mean baseline relative SCT at 3 months after PDT was significantly smaller than that in the PDT ineffective group (83.0 ± 1.4% vs. 97.6 ± 3.9%, *P* = 0.009).

### Multivariate analysis of parameters related with successful PDT and visual acuity 3 years after PDT

Multiple logistic regression analysis showed that unsuccessful PDT was significantly associated with age [odds ratio (OR) = 1.075, 95% confidence interval(CI) 1.007–1.147, *P* = 0.029)] and BCVA before PDT (OR = 14.5, 95% CI 1.4–157.7, *P* = 0.026). Multiple regression analysis showed that BCVA at 3 years was significantly associated with BCVA before PDT (β = 0.514, SE = 0.052, standardized β = 0.751, 95% CI for β = 0.411–0.617, *P* < 0.001).

## Discussion

In this long-term study, BCVA and age were the long-term prognostic factors of chronic CSC after half-dose PDT. Fujita et al. reported the 1-year results of half-dose PDT for chronic CSC. According to their study, the unsuccessful cases had significantly lower BCVA at the baseline than the successful cases.[26] BCVA was a prognostic factor for both the short-term and long-term periods. Age was not a prognostic factor for short-term period, but it was able to predict long-term outcome.

Our study suggested that relatively early PDT may improve outcomes of chronic CSC. Accumulation of serous retinal detachment (SRD) results in damage of photoreceptor cells,[27] retinal atrophy,[28] and RPE damage.[13] Aging is associated with decreased RPE cell density,[29] decreased Arden ratio, and increased time to light peak in the electrooculogram.[30] These reports suggested that accumulation of SRD and aging may cause reduction of RPE function. Inoue et al. reported that eyes without intense hyperfluorescence on ICGA were not responsive to PDT and that the recurrence rate was predicted to be high.[31] This suggested that PDT was effective in resolution of SRD only in cases with high hydrostatic pressure in the choroid. They also suggested that in eyes with CSC and intermediate hyperfluorescence, a disrupted RPE may not tolerate small changes in choroidal hydrostatic pressure, resulting in recurrences. SRD accumulation would occur when hydrostatic pressure in the choroid exceeds RPE tolerance. PDT induces occlusion of the choriocapillaries[32] and reduces hydrostatic pressure in the choroid. As PDT does not restore disrupted RPE, it is not effective in cases with disrupted RPE and intermediate hydrostatic pressure. These findings and our results suggested the importance of relatively early treatment of PDT, before the disruption of RPE.

Our study showed that low BCVA at baseline resulted in low BCVA at 3 years after half-dose PDT. Another long-term study on CSC treated by half-dose PDT showed no correlation between preoperative BCVA and final BCVA.[25] This result was inconsistent with our result. This may be due to the difference in background of the patients. In their study, complete resolution of SRD was observed in all 56 (100%) eyes, the subjects were much younger with a mean age of 45 years, and the genetic background might have been different. Previous short-term studies also showed a significant correlation between the baseline and final BCVA values. [26, 33] Ooto et al. reported that damage of photoreceptor cells was associated with loss of BCVA.[27]Damage of photoreceptor cells may not be restored even after a prolonged period after half-dose PDT. A relatively early half-dose PDT before the occurrence of visual impairment is important to maintain good visual acuity in eyes with CSC.

The choroid of the eyes with CSC is thicker than that of normal eyes.[10] Previous studies have shown that the choroidal thickness rapidly decreased after successful PDT and that it remained decreased for a year. [14, 34–36] To the best of our knowledge, our study was the first to demonstrate the changes in choroidal thickness of eyes with CSC after PDT in the long term. Choroidal thickness rapidly decreased after PDT, then gradually decreased for the long-term. The decrease in choroidal thickness from 12 months to 36 months after PDT was 8.2 μm in the successful group. This was very consistent with the results of a previous study on normal eyes, which reported that choroidal thickness decreased by 4.1 μm for every year increase in age.[37] Therefore, gradual decrease of choroidal thickness may be caused by an aging effect, during which the effect of the PDT may no longer exist. Further study is needed to find the long-term changes in choroidal thickens after PDT.

Uetani et al. previously reported that choroidal thickness remained unchanged after unsuccessful PDT. [38] Our study also showed small changes in SCT in eyes with persistent SRD. These results suggested that decreasing the thickness of the choroid may be the key factor in treating CSC.

In this study, SRD was initially resolved after one session of half-dose PDT in 74 (94%) eyes; of these, 3 (4%) had recurrence within 1 year. In earlier studies on more than 20 eyes with chronic CSC that were followed-up after more than 1 year, the rate of SRD resolution after half-dose PDT was reported to be 83% to 100%, whereas the recurrence rate within 1 year was reported to be 0% to 10%.[22, 24–26] These results were comparable to those of our study. Our results showed that SRD recurrence continued to occur during the long-term follow up period;10 (14%) eyes had recurrence within the entire follow-up period of more than 3 years (Fig 1). Therefore, long-term follow up is necessary for early detection of recurrence after complete SRF absorption.

This study had several limitations, including its retrospective and nonrandomized design. It also carried a selection bias on subjects with poor prognosis, because the design had a long follow-up period. The duration of symptoms was not added to the independent variables for the analysis because some patients have a long history of CSC and did not remember the exact duration of symptoms.

In conclusion, half-dose PDT for chronic CSC had high anatomical and functional success rates in the long-term follow-up period of more than 3 years. BCVA and age were the long-term prognostic factors of chronic CSC treated by half-dose PDT. Low BCVA and old age were associated with recurrence and persistence of SRD. In addition, low baseline BCVA resulted in low BCVA at 3 years after half-dose PDT. Relatively early half-dose PDT is recommended in order to maintain good visual function of patients with chronic CSC.

## Supporting information

S1 Table Dataset of patients. (PDF)
