# Supplementary material for: Long-term prognostic factors of chronic central serous chorioretinopathy after half-dose photodynamic therapy: A 3-year follow-up study
Source: PLoS One. 2017 Jul 24;12(7):e0181479. doi: 10.1371/journal.pone.0181479 (PMC5524407; doi:10.1371/journal.pone.0181479)
Supplement: S1 Table — (PDF) [file pone.0181479.s001.pdf]

	previous or concurrent use of corticosteroids	History of smoking	Refractive error (SE, diopters)	BCVA at baselkine (logMAR)	BCVA 3 years after PDT (logMAR)	Central foveal thickness (μm)	Neuroretinal thickness (μm)	Subretinal fluid height (μm)	Subfoveal choroidal thickness (μm)	PDT spot area (mm^2)	Window defect area (mm^2)
Unsuccessful group 1	No	No	−1.500	0.30103	0.22185	317.0	217.5	99.5	442.5	15.9	2.7
Unsuccessful group 2	No	Yes	−1.125	0.22185	0.00000	213.0	162.5	50.5	178.0	4.3	1.1
Unsuccessful group 3	No	No	−0.250	0.22185	0.09691	449.0	209.5	239.5	512.5	6.8	5.7
Unsuccessful group 4	No	Yes	−0.625	0.22185	0.00000	242.5	178.0	64.5	380.5	3.6	1.7
Unsuccessful group 5	No	No	−0.125	0.79588	0.30103	197.5	112.0	25.0	458.0	7.8	6.2
Unsuccessful group 6	No	No	1.875	0.30103	0.22185	243.0	160.0	83.0	358.0	36.0	20.5
Unsuccessful group 7	No	No	−1.125	0.22185	0.09691	226.0	142.0	84.0	443.0	4.0	0.1
Unsuccessful group 8	No	Yes	−2.125	1.00000	0.69897	259.5	122.0	137.5	397.5	13.2	4.1
Unsuccessful group 9	No	Yes	0.750	0.22185	0.00000	437.0	130.0	307.0	496.0	13.8	15.9
Unsuccessful group 10	Yes	No	−0.125	0.22185	0.00000	184.0	184.0	0.0	273.5	5.7	4.8
Unsuccessful group 11	Yes	Yes	0.250	0.30103	0.09691	233.0	172.5	60.5	295.5	12.6	4.4
Unsuccessful group 12	Yes	Yes	0.500	0.09691	0.09691	470.5	188.0	282.5	289.0	17.3	1.8
Unsuccessful group 13	No	No	4.000	0.22185	0.09691	520.0	150.5	369.5	440.0	17.3	24.6
Unsuccessful group 14	Yes	No	−4.375	0.09691	−0.11394	252.0	165.5	81.5	272.0	11.9	0.2
Unsuccessful group 15	No	Yes	−2.875	0.39794	0.39794	240.0	204.0	36.0	260.0	3.3	0.3
Successful group 1	No	Yes	0.500	0.00000	−0.11394	364.0	184.0	180.0	419.5	8.0	0.3
Successful group 2	No	No	−0.500	0.22185	0.00000	320.0	173.0	147.0	440.0	20.4	3.5
Successful group 3	No	Yes	−4.750	0.09691	0.00000	242.0	191.5	50.5	422.0	5.5	2.7
Successful group 4	No	Yes	0.875	0.09691	0.00000	375.5	148.5	227.0	503.0	8.8	3.0
Successful group 5	No	Yes	0.750	0.09691	0.00000	255.0	151.5	103.5	592.0	13.9	32.6
Successful group 6	No	No	0.500	0.00000	−0.11394	500.5	199.5	301.0	418.5	4.7	1.0
Successful group 7	Yes	Yes	−0.250	0.00000	0.09691	307.0	198.5	108.5	404.5	19.4	4.5
Successful group 8	No	No	−0.250	0.00000	0.00000	466.5	189.5	277.0	358.0	6.4	2.7
Successful group 9	No	Yes	2.500	0.22185	0.00000	347.0	147.0	200.0	475.0	8.0	4.7
Successful group 10	No	No	−1.250	−0.11394	0.00000	235.0	235.0	0.0	561.0	26.4	11.4
Successful group 11	No	Yes	−0.250	−0.07918	−0.11394	287.5	219.5	68.0	514.0	7.2	0.7
Successful group 12	Yes	Yes	−1.125	0.22185	−0.11394	308.0	196.5	111.5	396.5	8.0	2.6
Successful group 13	No	Yes	2.000	0.22185	0.09691	562.0	196.0	366.0	331.0	4.7	0.9
Successful group 14	No	Yes	0.625	0.30103	0.22185	179.5	122.0	8.0	427.0	8.6	7.1
Successful group 15	No	Yes	0.750	0.30103	−0.11394	420.5	175.5	245.0	506.5	5.5	0.6
Successful group 16	No	No	−2.000	−0.11394	0.00000	347.0	205.0	142.0	374.5	4.3	0.6
Successful group 17	No	Yes	−5.250	0.30103	0.09691	218.5	115.5	103.0	437.0	17.4	10.3
Successful group 18	No	No	−0.500	0.09691	−0.11394	282.5	143.5	139.0	545.0	4.0	4.2
Successful group 19	No	Yes	−0.125	0.00000	−0.07918	338.5	209.5	129.0	354.5	16.6	3.2
Successful group 20	No	Yes	−1.750	0.22185	0.00000	432.5	219.0	213.5	250.0	13.2	2.1
Successful group 21	No	Yes	0.500	0.39794	0.30103	206.0	121.0	85.0	296.5	4.3	0.2
Successful group 22	Yes	Yes	−1.375	0.00000	0.00000	294.5	162.0	132.5	454.5	11.3	2.1
Successful group 23	No	Yes	−0.125	0.00000	−0.11394	304.5	185.5	119.0	298.0	9.9	9.7
Successful group 24	No	Yes	0.125	0.00000	0.00000	343.5	224.5	119.0	399.5	11.6	5.7
Successful group 25	No	Yes	−0.375	−0.11394	0.00000	240.5	240.5	0.0	647.5	39.6	6.0
Successful group 26	No	Yes	−1.000	1.00000	0.09691	383.5	213.0	170.5	863.0	8.3	0.4
Successful group 27	No	Yes	−0.375	0.09691	0.00000	162.5	125.0	37.5	396.5	18.1	16.4
Successful group 28	No	Yes	0.000	0.09691	−0.11394	482.5	200.5	282.0	804.5	21.2	6.3
Successful group 29	Yes	Yes	−3.000	0.39794	0.22185	312.0	159.0	153.0	303.5	5.7	3.0
Successful group 30	No	Yes	−0.500	0.22185	0.04576	218.0	123.5	94.5	263.5	7.1	0.1
Successful group 31	No	Yes	−0.500	0.00000	−0.11394	308.5	193.5	115.0	525.5	4.0	3.2
Successful group 32	No	Yes	−0.625	0.79588	0.39794	419.5	140.0	279.5	430.5	15.2	3.9
Successful group 33	No	Yes	−5.000	0.09691	0.00000	308.5	223.0	85.5	498.0	9.9	0.5
Successful group 34	No	No	0.500	0.39794	0.22185	294.5	192.0	102.5	558.0	7.8	0.5
Successful group 35	No	No	−0.375	0.30103	0.09691	313.0	170.0	143.0	421.5	43.0	12.3
Successful group 36	No	Yes	4.000	0.09691	0.00000	295.0	204.0	91.0	400.5	15.2	9.4
Successful group 37	No	Yes	−0.375	0.22185	0.00000	242.0	166.5	75.5	214.5	6.2	3.4
Successful group 38	No	No	1.375	0.22185	0.00000	302.5	189.5	113.0	307.5	7.1	0.6
Successful group 39	No	No	0.250	0.04576	0.00000	405.5	333.0	72.5	382.5	5.7	2.5
Successful group 40	No	Yes	−0.500	0.30103	0.00000	282.5	171.5	111.0	388.5	6.2	1.7
Successful group 41	No	No	2.250	0.09691	0.00000	449.5	204.0	245.5	346.5	6.8	1.1
Successful group 42	No	Yes	−0.500	0.09691	0.09691	268.5	126.5	142.0	425.5	16.6	2.3
Successful group 43	No	No	0.000	0.30103	0.00000	539.5	178.5	361.0	222.0	29.1	15.7
Successful group 44	No	Yes	−0.625	0.09691	0.00000	317.0	188.0	129.0	501.0	7.1	2.3
Successful group 45	No	Yes	−1.250	0.15490	0.30103	309.5	128.0	181.5	366.5	3.5	6.3
Successful group 46	No	Yes	−1.125	0.00000	−0.07918	223.5	184.5	39.0	295.5	5.7	2.2
Successful group 47	Yes	Yes	−1.250	0.30103	0.22185	371.0	177.5	193.5	345.0	5.3	1.5
Successful group 48	No	Yes	−4.250	0.00000	0.00000	367.5	213.0	154.5	251.5	3.0	0.3
Successful group 49	No	No	0.125	0.00000	0.00000	418.0	179.0	239.0	606.5	38.5	14.6
Successful group 50	No	Yes	0.250	0.69897	0.30103	429.5	146.5	283.0	449.5	19.6	0.9
Successful group 51	No	Yes	−0.375	0.09691	0.00000	319.0	186.0	133.0	548.0	12.4	24.4
Successful group 52	Yes	No	0.125	0.22185	0.22185	382.0	145.5	236.5	306.5	16.1	3.8
Successful group 53	No	No	−3.250	0.00000	0.00000	422.5	222.0	200.5	346.5	4.2	1.2
Successful group 54	No	No	−0.625	0.22185	0.09691	257.0	211.5	45.5	217.0	9.6	4.2
Successful group 55	No	Yes	−3.500	0.39794	0.00000	253.0	133.0	120.0	511.0	16.6	11.2
Successful group 56	No	Yes	0.750	0.00000	−0.11394	338.5	180.0	158.5	369.5	18.1	9.4
Successful group 57	No	Yes	3.375	0.60206	0.52288	185.0	135.0	50.0	679.0	26.2	38.1
Successful group 58	No	No	−0.500	0.00000	0.00000	337.0	196.5	140.5	571.0	6.8	5.7
Successful group 59	No	No	1.250	0.52288	0.39794	273.5	123.5	150.0	336.5	5.1	Unmeasurable
Successful group 60	No	Yes	−1.125	0.30103	0.22185	361.5	169.0	192.5	388.5	5.9	0.1
Successful group 61	No	Yes	0.875	0.00000	0.00000	438.5	148.5	290.0	515.0	18.1	14.9
Successful group 62	Yes	Yes	0.375	0.69897	0.39794	271.5	191.5	80.0	437.0	13.9	8.4
Successful group 63	No	Yes	−1.375	0.09691	0.00000	258.5	199.0	59.5	634.5	47.3	45.7
Successful group 64	No	Yes	0.375	0.00000	0.00000	200.5	162.0	38.5	350.0	4.7	0.6
